# Virtual Tools to Enable Management of Knee Osteoarthritis

**DOI:** 10.1007/s40674-023-00202-2

**Published:** 2023-03-11

**Authors:** Rana S. Hinman, Belinda J. Lawford, Rachel K. Nelligan, Kim L. Bennell

**Affiliations:** Centre for Health, Exercise and Sports Medicine, Department of Physiotherapy, School of Health Sciences, Faculty of Medicine Dentistry & Health Sciences, The University of Melbourne, Melbourne, VIC Australia

**Keywords:** Knee osteoarthritis, Telehealth, Exercise, Digital health, mHealth, Internet

## Abstract

**Purpose of review:**

There is increasing recognition that virtual tools, enabled by the internet and telecommunications technology, can increase access to health care. We review evidence about the clinical effectiveness and acceptability of telephone-delivered and videoconferencing clinician consultations, websites and internet-delivered programs, and SMS and mobile applications in enabling the management of people with knee osteoarthritis (OA). We discuss barriers to using virtual tools and suggest strategies to facilitate implementation in clinical settings.

**Recent findings:**

An increasing number of systematic reviews, meta-analyses, and clinical trials provide evidence showing the effectiveness of virtual tools for improving knee OA management. Qualitative research shows that virtual tools increase patient access to knee OA care, are generally acceptable and convenient for patients, but can be associated with barriers to use from patient and clinician perspectives.

**Summary:**

Virtual tools offer new opportunities to enable people with knee OA to manage their condition and receive care that may otherwise be difficult or not possible to access. Telephone calls and videoconferencing can be used for real-time synchronous consultations between clinicians and patients, increasing the geographic reach of health services. Websites and internet-based programs can be used to educate patients about their condition, as well as deliver exercise, weight management, and psychological interventions. Mobile apps can monitor and track OA symptoms, exercise, and physical activity, while SMS can facilitate positive behaviour changes for self-management over the long-term when sustained clinician contact may not be possible.

## Introduction

Knee osteoarthritis (OA) is a major contributor to global disability [1] and the disease burden is set to escalate in the future [2, 3] as OA prevalence increases with the ageing of the population and rising obesity rates. It will become increasingly difficult for health services to manage people experiencing persistent knee pain and physical dysfunction typically associated with knee OA, and innovative approaches to delivering care at scale will be required. Clinical practice guidelines for knee OA emphasise the fundamental role of non-drug non-surgical strategies [4]. Self-management and patient-driven treatment options are the preferred approach, rather than clinician-delivered passive therapies. Advice and educational information for self-management, exercise and physical activity, and weight loss (for those who have overweight or obesity) are thus considered core management in all clinical guidelines [4] for people with knee OA.

People with knee OA may experience difficulty in accessing clinicians and healthcare services [5]. For example, people who live in rural or remote areas often experience geographical distance from health services or a shortage of health care professionals and/or services [6]. Even in metropolitan regions, travelling to visit a clinician in-person can be difficult for people who knee OA who experience restricted mobility outside the home [7]. For people with knee OA who are able to attend a health service in-person, consultations with a clinician may be limited by waiting lists and/or funding constraints [5]. People with knee OA often desire or require more sustained and/or frequent support from healthcare services to assist them in making the positive behavioural changes necessary to engage in exercise and weight loss over the long-term [8–10].

Evolving technology provides opportunities to increase the accessibility of healthcare services via ‘virtual’ strategies (often also termed telehealth, eHealth, mHealth, or digital healthcare). The European Alliance of Associations for Rheumatology (EULAR) advocates that remote care be considered in all parts of the patient pathway for self-management interventions for people with rheumatic and musculoskeletal diseases [11••]. The National Institute for Health and Care Excellence (UK) recommends digital and mobile health interventions be considered an option for any person who would benefit from improving their diet and/or increasing physical activity [12]. In particular, the guidelines advocate the important role of self-monitoring to review progress towards individual diet and physical activity goals. This review will focus on how virtual tools — including telephone and videoconferencing consultations, websites, internet-delivered programs, SMS, and mobile applications — may enable the management of people with knee OA. Challenges to using virtual tools in clinical practice will also be discussed.

## Videoconferencing consultations

Numerous recent systematic reviews have evaluated the effectiveness of virtual interventions for people with knee OA [13•, 14, 15••, 16, 17], collectively concluding that these models of service delivery can improve symptoms like joint pain and physical function. However, although videoconferencing consultations (i.e. synchronous video calls with a clinician using software such as Zoom, WhatsApp, Teams) are a common method for delivering virtual clinical care [18, 19], there is surprisingly little research evaluating its effectiveness in people with knee OA. For example, only one RCT included in those systematic reviews involved videoconferencing consultations. That trial found that an intervention involving seven individual consultations with a physiotherapist over Skype for a knee strengthening program, as well as access to an online pain coping skills program, led to clinically significant improvements in pain, physical function, and quality of life, compared to an information-only control group [20]. Importantly, those participants, as well as the physiotherapists who provided care, described overall positive experiences with videoconferencing consultations [21].

Since those systematic reviews [13•, 14, 15••, 16, 17], two new RCTs evaluating the effectiveness of videoconferencing consultations for people with knee OA have been published. One 3-arm trial compared two 6-month exercise programs delivered by physiotherapists via Zoom, one with and one without a weight loss dietary program administered by dietitians via Zoom, compared to information-only control [22•]. Both interventions led to improved pain and physical function, with the combined exercise plus diet program conferring modest benefits over exercise only. Both programs were also found to be cost-effective [23], and qualitative research showed that both patients and dietitians had positive experiences using videoconferencing to receive/deliver the weight loss program [8]. Another 3-arm RCT in people with knee OA and obesity utilised a blended approach to delivering resistance exercise and dietary care [24]. Participants received initial in-person consultations with dietitians or sports medicine clinical staff and were then followed up once a week for 12 weeks via telephone or videoconferencing. This RCT showed that, compared to diet or exercise alone, the combined diet plus exercise intervention led to greater improvements in physical function, reduced cholesterol, and reduced triglyceride levels. Collectively, the existing evidence suggests that exercise and weight loss programs delivered via videoconferencing with qualified health professionals are effective for people with knee OA. However, no clinical trials have evaluated the effects of videoconferencing consultations in comparison to traditional in-person consultations, although an RCT is underway [25].

There is growing evidence that performance-based tests of strength, physical function, and balance can be administered reliably by clinicians via videoconferencing. Such tests are important to allow clinicians to evaluate treatment response and adjust treatment if required. Recent systematic reviews found that several performance-based tests have sufficient reliability and agreement with in-person test scores when administered via videoconferencing [26, 27, 28••]. However, only four of the studies included in those reviews focused on telehealth assessment of people with chronic lower limb musculoskeletal conditions [29–32], and the overall evidence was of low to very low quality. Furthermore, most studies utilised expensive and/or complex videoconferencing systems, which limits the generalisability of findings to ‘real-world’ clinical settings. Since those reviews, a recent study [33] investigated the test–retest reliability, and the agreement with scores in-person, of performance-based tests that are recommended by the Osteoarthritis Research Society International for people with hip/knee OA [34]. That study utilised pragmatic methods (i.e. used freely available videoconferencing software and equipment found around the home) and found that many tests had acceptable test–retest reliability and acceptable agreement with scores obtained in-person (e.g. stair climb test, timed up and go, right leg timed single-leg stance, calf raise). However, some were found to have lower levels of reliability or agreement (e.g. fast-paced walk test).

## Telephone consultations

Compared to videoconferencing, more research has focused on telephone-delivered interventions for people with knee OA [13•, 14, 15••, 16, 17, 35]. Numerous RCTs have shown that telephone-delivered exercise can benefit people with knee OA. For example, prior trials have found no differences in clinical outcomes between physiotherapist-prescribed exercise programs delivered via telephone and delivered in-person, in terms of improvements in pain, function, and quality of life [36, 37]. Two RCTs have compared telephone-delivered exercise by a physiotherapist [38•] or a health educator [39] to information-only control groups, collectively reporting positive effects on pain and physical function post-intervention. However, as with many in-person delivered exercise programs, benefits were not sustained in the longer-term [38•]. Other exercise RCTs have evaluated interventions using a combination of in-person and telephone consultations. For example, two RCTs found that interventions involving initial in-person consultations of education and exercise, followed by telephone advice and support, led to greater improvements in physical activity compared to a wait list [40], and were no different to clinical outcomes following the same program delivered entirely in-person [41].

Beyond exercise, there is some evidence that other telephone-delivered interventions may benefit people with knee OA, including health/motivational coaching, cognitive behavioural therapy (CBT), and dietary weight loss. Two RCTs evaluated the effects of adding coaching/motivational telephone calls to promote behaviour change and long-term adherence during/after a prescribed exercise program [42, 43]. Although one study found that the telephone group had higher physical activity, more global improvements, and better exercise adherence immediately post-intervention [42], neither study found any evidence of effects on adherence in the longer-term [42, 43]. Telephone-delivered CBT may also have benefits for people with knee OA, with a recent RCT finding that a telephone-delivered CBT for insomnia in adults with OA led to improved sleep, fatigue, and pain, compared to information-only control [44•] and was cost-effective [45]. Multi-component interventions (i.e. involving a physical activity program, weight management advice, and CBT strategies), delivered via telephone by a counsellor, have also been found to lead to small improvements in physical function, compared to usual care [46, 47]. In contrast, telephone-delivered generic weight management and healthy lifestyle advice does not appear to be effective for knee OA, with one RCT showing no significant improvements in knee pain or body weight compared to usual care [48].

## Websites and online programs

The number of websites about OA is rapidly escalating [49], with many patients using the internet to seek information about their condition and treatment options and/or to undertake structured treatment programs for their OA symptoms. Various types of OA websites exist including academic/non-profit, physician, social, and commercial, the latter being the most common [50]. However, studies have shown that the quality, readability, and content of OA websites are variable [49, 51•], with differences between countries and between website types [50]. Of 27 freely available contemporary pain management websites identified in a scoping review, Arthritis Australia’s MyJointPain (which is specifically for OA), Pain Canada’s LivePlanBe, and the ACI Pain Management Network were the top ranked in terms of quality and best practice self-management support strategies [52]. Selected evidence-based website information/resources/programs for OA that may be of use in the clinical setting are outlined in Table 1.

**Table 1:** Selected examples of English-language evidence-based virtual websites/programs/applications that may be used to support the management of people with knee osteoarthritis (OA)

Virtual tool	Description	Supporting evidence
My Knee Exercise https://mykneeexercise.org.au	Website with instructions for progressive home-based strengthening exercises, as well as educational information and downloadable documents to support exercise adherence	RCT in 206 people with knee OA showed the strengthening program, when combined with SMS support, reduced knee pain, and improved function compared to a control website with educational information only [53•]
My Joint Yoga https://myjointyoga.com.au	Website with a 12-week yoga program delivered in pre-recorded video format with an instructor and group class, as well as educational information	RCT in 212 people with knee OA showed the yoga program plus online education improved function, knee stiffness, quality of life, and arthritis self-efficacy compared to online education alone [54•]
My Exercise Messages Apple store and Google play	Mobile app that allows tracking of completed weekly exercise sessions and provides regular messages to facilitate weekly exercise and personalised messages to help overcome individual barriers to exercise participation	Adapted from an RCT in 110 people with knee OA, which showed that the behaviour change messages delivered via SMS improved adherence to home exercise at 24 weeks in people with knee OA [55•]. The behaviour change messages were designed using the Behaviour Change Wheel framework and developed to address key barriers and facilitators to exercise participation in adults with hip and/or knee OA [56]
ESCAPE-pain https://escape-pain.org	Web program and app that supports the ESCAPE-pain program which is an in-person group rehabilitation program that integrates educational self-management and coping strategies with an individualised exercise regimen	A pragmatic program evaluation between 2014 and 2018 involving over 110 clinical and non-clinical sites and reaching over 9000 people with OA showed sustained clinical effectiveness and high levels of adherence within a range of real-world settings [57]
Walk with Ease Apple store and Google Play	Mobile app that outlines the US Arthritis Foundation’s 6 week Walk with Ease program. Used in conjunction with the booklet and a Fitbit	Longitudinal cohort evaluations of the program (in classes or self-directed) in various settings/populations have shown it can increase distance walked and improve symptoms and self-management [58–60]
PhysiTrack https://www.physitrack.com.au	Web platform and mobile app for remote care delivery, including exercise programming system with exercise videos and instructions/dosages delivered to the patient’s smartphone or computer	RCT in 305 people with acute or chronic musculoskeletal conditions showed that adherence to a physiotherapist-prescribed exercise program was greater when the web-based system was used compared with the therapists’ usual methods [61]
Joint Academy https://www.jointacademy.com/us/en/	Web and app program of structured OA information and individualised neuromuscular exercise, supervised by a physiotherapist via asynchronous chat and/or telephone. Available in USA only	A longitudinal cohort study in 499 people with knee and/or hip OA showed that continuously participating in the web-based program was associated with improvements in pain and function [62]. RCT in 105 people with knee OA showed the app program reduced knee pain compared to routine self-management [63]
My Joint Pain https://myjointpain.org.au	Website from Arthritis Australia which provides information about OA and treatments. Allows individuals to input information in order to receive a risk assessment, tailored management plan and weekly check-ups	Quasi-experimental design in 195 people with knee and/or hip OA showed significant improvements for users compared to non-users in self-management and weight reduction [64]
painTrainer www.paintrainer.org	Interactive program that teaches cognitive and behavioural strategies to manage chronic pain. The program comprises 8 weekly sessions each lasting 30–45 min	RCT in 113 people with knee and/or hip OA showed the program led to improvements in pain and self-efficacy compared with no treatment control [65]. RCT in 148 people with knee OA showed the program, when combined with videoconferencing consultations with a physiotherapist for home exercise, led to improved pain and function compared to internet information [20]
Somryst (previously SHUTi) https://www.somryst.com	Mobile app to treat insomnia in adult and elderly people. It is based on cognitive behavioural principles and involves a 6-week structured interactive fully automated intervention. Available in USA only	RCT in 303 adults with chronic insomnia showed improvements in various sleep indices which were maintained at 12-months, compared to online education control [66]
Better Knee, Better Me https://www.medibank.com.au/health-support/health-services/better-knee-better-me/	12-month program that includes exercise and pain management supported by a physiotherapist, and weight loss (with ketogenic very low calorie diet and meal replacements) supported by a dietitian, via videoconferencing consultations. Available to eligible members of Australian private health insurer Medibank	RCT in 415 people with knee OA and overweight/obesity showed that the exercise and diet program improved pain and function, as well as average weight loss of 9 kg [22•]
Healthy Weight for Life https://healthyweightforlife.com.au/	Digital program that includes a very low calorie meal replacement diet, exercise, and healthcare team to provide support via phone, SMS, email, and private online message board. Available in Australia only	Longitudinal cohort study of 1383 people with knee OA showed 94% of people achieved > 2.5% loss of body weight, with greater weight loss associated with greater improvements in pain and function [67]
This Way Up. The Depression Program https://thiswayup.org.au/programs/depression-program/	Online program with 6 online lessons of best practice cognitive behavioural therapy plus regular homework assignments, reminder emails/texts, and access to supplementary resources	RCT in 69 people with knee OA and comorbid major depressive disorder showed reductions in depressive symptoms and psychological distress compared to usual care [68]
Physiotherapy Exercise and Physical Activity for Knee Osteoarthritis (PEAK) https://www.futurelearn.com/courses/peak	Web-based 4-week educational course to guide physiotherapists in how to implement best-practice care to people with knee OA, delivered through one-to-one consultations, via videoconferencing (using the Zoom platform) or during face-to-face, in-person consultations. Contains downloadable patient resources and access to a video library of exercises. Also available in Spanish, Portuguese, and Chinese	Longitudinal cohort study in 1318 registrants completing the course showed increased confidence with videoconferencing and increased likelihood of using education, strengthening and physical activity in a knee OA treatment plan, compared to pre-course [69]. Qualitative study in 15 physiotherapists revealed positive experiences with the e-learning course and improved confidence with videoconferencing after course completion [70]
EduWeight: Weight Management for Adult Patients with Chronic Disease https://www.futurelearn.com/courses/eduweight	Web-based 6-week educational course for health professionals to improve their confidence and skills in helping their patients living with chronic diseases manage their weight. Contains downloadable patient resources for use in clinical setting	RCT in 80 physiotherapists showed that clinicians allocated to the education course had greater improvements in confidence, knowledge and skills about weight management, and reduction in weight stigma, compared to the control group [71]
My Knee https://myknee.trekeducation.org/	Website containing a toolkit of evidence-based information and tools to support people with knee OA make informed decisions about their care and improve their self-management	Based on a systematic review of knee OA education interventions [72] and concept mapping studies involving people with knee OA [73] and physical therapists [74] that identified broad educational needs and priorities

Several structured self-directed web-based exercise programs have been developed for, and tested in, RCTs in people with knee OA. The results have been somewhat variable, which may reflect differences in program duration, type of exercise, individualisation, and use of behaviour change techniques and adherence. One RCT tested a 9-week behavioural graded activity program in 199 people with knee and/or hip OA [75]. Inconsistent benefits for function and physical activity levels were found, but only 46% reached the adherence threshold of completing 6 out of the 9 modules, highlighting a lack of engagement. Another program incorporated tailored progressive exercises based on an algorithm and inputted patient data, video demonstrations, automated reminders, and progress tracking [76]. Engagement with this program was also relatively low and most clinical outcomes were not different from physiotherapist treatment or wait list control. Better adherence and outcomes were noted with a 24-week self-directed web program including a strengthening exercise regimen, physical activity guidance, and information, supported by automated behaviour change text messages [53•]. Finally, an online self-directed 12-week yoga program of pre-recorded videos improved function, knee stiffness, quality of life, and arthritis self-efficacy, but not knee pain, compared to online education alone in 212 people with knee OA [54•]. Overall, the results suggest that self-directed web-based exercise programs can have beneficial effects in people with knee OA, although lack of engagement can be an issue. Qualitative evaluations reveal that facilitators include ease of use, functionality, and development by a reputable source [77, 78]. However, some users deemed human interaction to be preferable [77, 78], highlighting that a blended approach with clinician input may enhance patient outcomes with these structured online programs. Another option is to use such exercise programs as the first step in a stepped care treatment model whereby patients only receive more intensive treatments, such as with clinician input, if they do not have clinically relevant improvements from the prior treatment step [79•].

Numerous dietary weight loss programs are available via the internet. A systematic review of web-based interventions exclusively for weight loss identified 11 RCTs in adults with overweight or obesity [80]. Results of a meta-analysis found moderate quality evidence that the web-based interventions were not different to the use of off-line interventions in terms of weight or body mass index. However, despite weight loss being a core recommended treatment for knee OA, to our knowledge, there is only one study reporting on a web-based program focused on weight loss in this patient population [81]. This 18-week program (Healthy Weight for Life) includes a very low-calorie diet with meal replacement supplements, activity and exercise plan, personalised online progress tracking (phone and mail alternatives available), and 2-way personal motivation, support, and advice via phone, short message service/text message, e-mail, message board, or mail from a member of the care support team. A longitudinal study of 1383 people with knee OA who completed this program showed a mean weight loss of 8% of baseline body weight and a dose–response relationship between amount of weight loss and improvements in symptoms [81].

Psychological interventions delivered via the web can be used to address mental health impairments, pain coping, and insomnia associated with knee OA. A meta-analysis of 70 RCTs of self-guided internet-based psychological interventions in people with a range of chronic health conditions found small effects in terms of reducing symptoms of depression, anxiety, and distress, with slightly stronger effects for CBT approaches and for those incorporating a clinician [82]. However, there are limited trials of such programs in people with OA [65, 68] and only one that specifically recruited a sample with depression [68]. In that RCT, a 10-week self-directed web-based CBT program targeting depression in people with knee OA and comorbid major depressive disorder showed large improvements in depressive symptoms and distress post-intervention and at the 3-month follow-up compared to usual care [68]. Most intervention participants (84%) no longer met depression diagnostic criteria at follow-up. Further benefits of the CBT program included improved self-efficacy, pain, stiffness, and physical function. An 8-week automated online pain coping skills training program [65], translated from an effective in-person therapist-delivered program for knee OA [83], led to lower pain as well as increased self-efficacy for pain management compared to no treatment in people with knee OA. While there are many web-based CBT programs for insomnia, none have been tested for their efficacy in people with knee OA. However, there is systematic review and meta-analysis evidence from the general adult population that self-directed internet CBT programs that typically include cognitive restructuring, sleep restriction, stimulus control, relaxation, and sleep hygiene education are effective treatments for insomnia [84], whether this would lead to simultaneous improvements in pain in knee OA is not known, although this was not found for in-person CBT for insomnia [85].

## Mobile applications (apps)

A systematic search of app stores identified 94 smartphone apps relevant to knee and/or hip OA management [86•]. However, most lacked evidence to support their design, usability, and effectiveness. Considering this, it is unsurprising that apps that are designed specifically for OA are of lower quality with lower potential for behaviour change compared to apps for other chronic conditions [87]. Although features vary, apps to support OA self-management typically prescribe and/or monitor exercise and physical activity, support adherence (e.g. exercise reminders via notifications, visual comparisons of actual exercise completed versus exercise goals), and/or track joint symptoms (e.g. pain, stiffness, physical function).

Of the few apps that have been evaluated in RCTs in knee OA, results appear promising [63, 88, 89, 90•]. A systematic review of RCTs comparing app-delivered therapeutic exercise to exercise delivered via other modes (e.g. paper-based handouts) for musculoskeletal conditions showed app use reduced pain and improved physical function, particularly in people with knee OA [90•]. Similarly, an RCT that evaluated 6-week use of Joint Academy, a commercially available app that contains hip/knee OA education, exercise prescription, and physiotherapist contact via asynchronous chat, resulted in greater pain reduction in people with knee OA compared to a usual care control [63]. Another RCT evaluated a mobile app, OA GO, coupled with a wearable activity tracker [89]. The app provided a visual display of daily step count and prompted daily self-report of joint pain and mood. This study found participants using the activity tracker supported by the app had a greater increase in steps per day compared to participants using a blind activity tracker (i.e. no access to steps recorded by the tracker) at 90 days (mean increase 1199 steps/day compared to 467). As each additional 1000 steps/day has been linked to 16–18% reduction in incident functional limitation in knee OA over 2 years, these findings may be clinically relevant [91]. Finally, an RCT is underway evaluating if a freely available app, My Exercise Messages, can enhance clinical outcomes from physiotherapist-prescribed home exercise in people with knee OA [92]. The My Exercise Messages app was designed to support adherence to exercise and physical activity in people with hip/knee OA by tracking weekly exercise sessions, delivering regular notifications to facilitate weekly exercise and provides personalised messages to help overcome individual barriers to exercise participation, if encountered. App development was based on a behaviour change text message program [56] found to improve adherence to home exercise in people with knee OA [55•]. Table 1 presents a selection of evidence-informed mobile apps for knee OA.

## Short Message Services (SMS)

Mobile phone usage is high in both advanced and emerging economies [93], making SMS or mobile phone text messaging a widely accessible technology with fewer barriers to engagement than other forms of digital communication. For example, SMS does not require expensive technology (e.g. smartphone), internet access, nor rely on technological literacy. SMS interventions have been found effective in the promotion of a range of health behaviours relevant to people with OA (e.g. physical activity, diet, and/or weight loss) [94–96]; however, few RCTs have evaluated SMS interventions specifically in knee OA [53•, 55•, 97]. One RCT showed that a 24-week tailored SMS intervention improved adherence to physiotherapist-prescribed home exercise at 24 weeks in people with knee OA [55•]. The automated SMS intervention was designed using the Behaviour Change Wheel framework and was developed to address key barriers and facilitators to exercise participation in adults with knee OA [56]. The same SMS intervention, combined with an unsupervised web-based strengthening exercise program, was evaluated in another RCT showing this combination improved knee OA clinical outcomes at 24 weeks, compared to an education control [53•]. A nested qualitative study showed that participants viewed the SMS intervention as a valuable exercise reminder that promoted accountability to the unsupervised exercise program [77]. Another RCT evaluated a psychological intervention delivered via automated SMS in people who had hip or knee joint replacement surgery postponed due to the COVID-19 pandemic [97]. It found that the intervention (two SMS per day for 14 days to encourage pain coping) led to meaningful clinical improvements at 2 weeks, compared to no contact.

## Challenges with virtual care

Barriers to implementing and engaging with virtual care exist and may explain why uptake was slow prior to the COVID-19 pandemic until public health orders and lockdowns forced a rapid shift to virtual care models. A systematic review of qualitative studies has explored the barriers from the patient’s perspective amongst people with chronic pain, including OA [98•]. In this review, patients found virtual care to be impersonal if they were unable to develop a rapport with a clinician or there was no clinician contact. Lack of physical presence, inability of clinician to touch, and limited nonverbal communication were all viewed as contributors to a poor rapport. Patients also felt that telehealth was impersonal when interventions were delivered inflexibly with limited scope for individualisation. Lack of cultural tailoring (e.g. use of American accents in an online program delivered to Australian participants [99]) was also perceived as a barrier to engagement. Technological challenges, such as poor internet connection or malfunctioning app- or web-based programs, were found to be a barrier to videoconferencing in particular. A mismatch between patient needs and expectations of virtual care and the actual components/content of the virtual intervention (e.g. irrelevant or unhelpful content, complex activities or information overload) can adversely affect patient motivation and engagement. Finally, limited digital literacy and lack of familiarity with the technology can also adversely hamper patient engagement with virtual interventions. Table 2 summarises important patient-level barriers to virtual care and outlines potential strategies for clinicians to overcome these.

**Table 2:** Patient barriers associated with implementation of, and engagement with, virtual care for knee osteoarthritis and potential solutions

Barrier	Potential solutions
Reduced sense of rapport with clinician	Prioritise videoconferencing over telephone for synchronous consultations to enable visual as well as verbal contact Consider asynchronous clinician check-in/follow-up with patients who have been recommended self-directed web- or app-based self-management programs Consider a blended approach to care (e.g. combining in-person and virtual care, synchronous clinician telehealth consultations with web-/app-based self-directed programs), where necessary
Perception that intervention is not individualised	Consider a blended approach to care — for example, an in-person visit first for thorough assessment — before implementing virtual intervention strategies Consider use of electronic patient-reported outcome measures and functional performance-based measures (synchronous or asynchronous via video recording) which can be administered/observed remotely Combine generic/less individualised online programs with individualised/more personal advice for home-based self-management
Technological difficulties/failures	Ensure clinician has optimal software and hardware for virtual consultations to minimise disruptions at the clinician end Educate patients how to optimise their internet speed/connection at home and set up their environment for optimal audio/video during consultations Provide clear instructions to the patient on how to set up and use the chosen virtual platform/application Clinician should be familiar with trouble-shooting solutions to assist the patient during synchronous virtual consultations
Mismatch between expectations and actual intervention	Educate patients about what specific virtual tools can and cannot offer them Ensure patient has choice in their treatment plans and provide adequate information about the advantages and disadvantages about all in-person and virtual treatment options so that they can make an informed decision
Reduced digital literacy	Determine patient confidence and competence with using the selected virtual tool prior to recommending it Encourage family member/carer support/assistance where feasible Follow-up with the patient to monitor engagement/use of recommended virtual tools and adjust treatment plans when necessary
Privacy and data security concerns	Ensure all patient and clinician devices used for virtual care have up-to-date and high-quality anti-virus protection and use firewalls Ensure Wi-Fi networks used for virtual care are secure and password-protected using strong passwords Only use/recommend mobile apps that are developed from credible organisations and that are available from reputable app stores (e.g. Google play, Apple’s App Store)
Concerns about trustworthiness of online information	Recommend and encourage use of high-quality websites, preferably those with Health on the Net code (HONcode) certification and from academic/non-profit sources

Clinicians must also be accepting of, and willing to use, virtual tools for successful implementation of virtual care models. Clinicians are often not confident or familiar with conducting synchronous telehealth consultations [100••] and are sceptical about their effectiveness [100••, 101], which may contribute to a reluctance in implementing virtual care. Core capability frameworks [102, 103] for telephone- and video-delivered care have been developed to assist clinicians to identify areas of their own practice that may benefit from up-skilling and participation in professional development can help improve confidence and knowledge about delivery of videoconferencing consultations [69]. Clinicians often have reservations about their ability to establish therapeutic alliance virtually [101], despite research evidence suggesting a strong therapeutic alliance is possible [21, 104]. Clinicians are also uncomfortable with the inability to touch the patient [100••, 105], perceiving this as a barrier to effective diagnosis and effective treatment.

Although clinicians consider online resources to be a useful adjunct to support pain self-management [106], they have concerns about the potential for misinterpretation and misinformation, inadequate quality control and evidence-based information, insufficient comprehensiveness, and lack of individualization. These concerns are particularly relevant given that many people with knee OA will access information from the web independently and without consultation of or discussion with a health professional. While online OA information quality has improved over the last two decades, the mean quality is still only considered ‘fair’ and a large number of websites exceed the recommended 7–8th grade readability level [49, 107]. For example, an analysis of accessibility of online self-management support webpages for people with OA showed that only 5 of 49 eligible webpages met the recommended reading level for health education literature [107]. For online knee OA information, research shows that few websites provide accurate and clear content aligned to important research evidence and that there is large variation in comprehensiveness and credibility [51•]. It is therefore important that clinicians develop and/or direct patients to readable, high-quality websites, preferably those with Health on the Net code (HONcode) certification and from academic/non-profit sources, as these are associated with better quality [49, 50].

Although beyond the scope of this paper, there are also system-level challenges with implementing virtual care. Barriers include restrictive funding models that do not necessarily reimburse telehealth-delivered care, patient privacy regulations, and geographical restrictions on clinician licensing [108]. While costs of delivering virtual/blended care may be lower than conventional care models [109], such care may not necessarily be more cost-effective from a societal or healthcare perspective. Although virtual care strategies have the potential to reduce healthcare inequalities in some groups (e.g. minority ethnic groups [110]), there is a risk that they can exacerbate inequality for others (e.g. people with low education or unemployed [110]). In low- and middle-income countries, system-level barriers to virtual care include unavailability of infrastructure (e.g. weak or slow internet connection), absent or unclear policies/regulations related to virtual care, and limited digital literacy in healthcare users [111].

## Conclusions

It is increasingly difficult for people to access healthcare for knee OA. Evidence-based management requires people with knee OA to make behavioural changes to follow health advice and actively self-manage their condition using exercise, weight control, and psychological strategies. However, there is limited capacity in health services to provide people with knee OA the ongoing support needed to maintain positive behavioural changes and engage with these interventions over the long-term. An increasing number of systematic reviews, meta-analyses, and clinical trials provide evidence supporting the effectiveness of virtual tools in the management of knee OA. Qualitative research shows that virtual tools increase patient access to knee OA care, are generally acceptable and convenient for patients, but can be associated with barriers to use from patient and clinician perspectives. Clinicians should recognise the important role that virtual tools can play in the long-term management of knee OA and consider incorporating these, as appropriate, into the care of individual patients.
